# Mechanisms of tumor necrosis factor-α and interleukin-6 induction during human liver transplantation

**DOI:** 10.1155/S0962935193000420

**Published:** 1993

**Authors:** Gerhard Hamilton, Sonja Vogel, Reinhold Fuegger, Franz X. M. Gnant

**Affiliations:** Department of Surgery University School of Medicine Alserstr. 4 Vienna A-1090 Austria

## Abstract

In human orthotopic liver transplantation (LTX) intraoperative elevations of TNF-α (> 100 pg/ml) and IL-6 (>800 pg/ml) have been found to correlate with early post-operative rejections and infections respectively. In this study the possible mechanism responsible for the induction of these cytokines has been investigated during liver allografting in 38 recipients. Intraoperative elevations of TNF-α (> 100 pg/ml) were detected in the majority of pre-transplant endotoxin positive recipients (8/12, > 10 endotoxin units/ml), the patients turning endotoxin positive until the end of grafting (3/5), and in a subgroup (6/21 patients), apparently endotoxin negative for the whole operation. Therefore endotoxin (ET) seems to stimulate release of TNF-α in approximately 50% of the patients, whereas sensitized Kupffer graft cells or immediate allograft reactivity of the host are likely to account for the remaining TNF-α positive cases. Elevations of IL-6 > 800 pg/ml) were found in approximately 50% of the TNF-α positive cases, indicating partially independent regulatory pathways for IL-6 induction in the TNF-α negative patients. In agreement with a previous study, 11/13 (85%) of the intraoperative TNF-α positive recipients rejected their grafts within the first 10 days post-operatively. These data demonstrate that ET/infection associated as well as ET independent/reperfusion associated intraoperative TNF-α elevations, promote the initiation of allograft rejection in human liver transplantation. The transient and low endotoxaemia caused by the liver grafting procedure performed without veno-venous bypass seems to be of minor importance in the intraoperative induction of TNF-α.


Mediators of Inflammation 2, 303–307 (1993)

### Mechanisms of tumor necrosis factor-α and interleukin-6 induction during human liver transplantation

Gerhard Hamilton[CA], Sonja Vogel, Reinhold Fuegger and Franz X. M. Gnant

Department of Surgery, University School of Medicine, Alserstr. 4, A-1090 Vienna, Austria

[CA] Corresponding Author

**In human orthotopic liver transplantation (LTX) intraoperative elevations of TNF-α (>100 pg/ml) and IL-6 (>800 pg/ml) have been found to correlate with early post-operative rejections and infections respectively. In this study the possible mechanism responsible for the induction of these cytokines has been investigated during liver allografting in 38 recipients. Intraoperative elevations of TNF-α (>100 pg/ml) were detected in the majority of pre-transplant endotoxin positive recipients (8/12, >10 endotoxin units/ml), the patients turning endotoxin positive until the end of grafting (3/5), and in a subgroup (6/21 patients), apparently endotoxin negative for the whole operation. Therefore endotoxin (ET) seems to stimulate release of TNF-α in approximately 50% of the patients, whereas sensitized Kupffer graft cells or immediate allograft reactivity of the host are likely to account for the remaining TNF-α positive cases. Elevations of IL-6 (>800 pg/ml) were found in approximately 50% of the TNF-α positive cases, indicating partially independent regulatory pathways for IL-6 induction in the TNF-α negative patients. In agreement with a previous study, 11/13 (85%) of the intraoperative TNF-α positive recipients rejected their grafts within the first 10 days post-operatively. These data demonstrate that ET/infection associated as well as ET independent/reperfusion associated intraoperative TNF-α elevations, promote the initiation of allograft rejection in human liver transplantation. The transient and low endotoxaemia caused by the liver grafting procedure performed without veno–venous bypass seems to be of minor importance in the intraoperative induction of TNF-α.**

**Key words:** Endotoxin, Induction of cytokines, Interleukin-6, Liver transplantation, TNF-α

### Introduction

Orthotopic liver transplantation is an accepted therapy for end-stage liver disease with high graft survival in properly selected patients.[1] However, primary nonfunction of the graft, frequent rejections, post-operative multi-organ complications, infections and further complications caused by the underlying diseases are observed in the majority of the recipients.[2] With the exception of primary nonfunction, surgical problems and acute drug toxicity, the diverse biological and pathological responses in patients with post-operative complications are correlated with elevated systemic and local levels of cytokines, as TNF-α, IL-1, IL-6, IL-8 and others.[3] Clinical and animal experimental liver transplantations are associated with systemic circulation of intestine derived endotoxin during recirculation after the anhepatic phase, possibly resulting in release of TNF-α and various other kinds of cytokines and toxic mediators from Kupffer cells and alveolar macrophages.[4] High peripheral blood concentrations of TNF-α were found in clinical allograft rejection, septic shock and hepatopulmonary damage in experimental animal models.[5] Anti-TNF-α antibodies were demonstrated to prolong graft survival, reverse rejections and to diminish the hepatic and pulmonary damage, providing clear evidence for the direct functional participation of TNF-α in graft damage and inflammation.[5,6]

In a previous study we measured the courses of ET, TNF-α and IL-6 plasma concentrations during human liver transplantation and correlated intraoperative elevations of TNF-α with subsequent rejections and those of IL-6 with post-operative infectious complications in a high percentage of the recipients in a multivariate analysis.[7,8] Furthermore detailed analysis of IL-6 in the pulmonal and radial artery and the femoral vein during LTX revealed the preferential production of IL-6 in resident lung macrophages in patients who developed infections in the early post-operative phase.[9] In these studies no direct correlation of intraoperative endotoxaemia with post-operative complications was observed.

© 1993 Rapid Communications of Oxford Ltd

In the present study the role of endotoxin in the induction of TNF-α and IL-6 was investigated in a larger group of liver transplant recipients. Although in selected animal models, ET was demonstrated to induce the typical pathophysiological changes associated with the liver grafting procedure, the situation in clinical liver transplantation with patients of widely different qualitative and quantitative liver impairment, variable length of the anhepatic phase, variations in MHC disparity and variable organ conservation, is expected to be much more complex.

### Patients, Materials and Methods

*Patients:* Thirty-eight orthotopic liver transplantations (LTX; two retransplantations) in 38 patients carried out at our institution between August 1990 and June 1991 were included in this study. The mean age of the patients (twelve females, 26 males) was 47 ± 14 years (range, 16–67 years) and indication for LTX was tumour in six (hepatocellular carcinoma, liver metastases), fulminant hepatitis in one, primary biliary cirrhosis in five, alcoholic liver cirrhosis in nine, Wilson's disease in two, cirrhosis (cryptogenic, post-hepatic and secondary biliary) in 13, and retransplantation after graft failure due to rejection in two cases. All recipient–donor pairs were matched for blood group compatibility, but not for their major histocompatibility antigens (two to three HLA mismatches). Liver grafting was performed as described previously.[7] Immunosuppressive therapy was initiated with methylprednisolone at the end of the operation, followed by an anti-thymocyte globulin preparation (ATG; Bio-Merrieux, France) preparation beginning on the second day post-transplant (200 mg/day) and finally after 8 days by cyclosporine/prednisolome maintenance therapy (aimed at 150–250 ng/ml CsA blood trough level as measured by HPLC; prednisolone tapered from 200 mg/day to 40 mg/day). Diagnosis of infection was based on bacteriological cultures from blood samples, urine, bile or ascites for bacterial infections and on virus isolation cultures from blood, urine and sputum and serology for viral infections. Rejections were diagnosed by clinical signs, increases in blood concentrations of bilirubin, liver enzymes and histological examination of liver biopsies (grade I–IV).

During transplantation, blood samples (3–5 ml) were drawn into heparinized tubes at the following time points: (a) immediately before operation; (b) before clamping of liver vessels; (c) at the beginning of the anhepatic phase; (d) at end of the anhepatic phase; (e) 5 min after recirculation; and (f) at the end of the operation. Blood was collected from the radial artery for all time points. Duration of the anhepatic phase was approximately 65 min, ranging from 45–145 min. The blood samples were stored at 4°C, centrifuged and the plasma fraction used in all determinations.

*Measurement of endotoxin:* Endotoxin was determined using a commercial Limulus amoebocyte lysate test system (Coatest Endotoxin; Kabi Diagnostica, Nykoepping, Sweden; *E. coli* 0111:B4 endotoxin standard) and a sample pretreatment method according to Berger *et al.* with modifications.[10] In brief, 0.5 ml plasma was centrifuged in Centrisart I (Sartorius, Goettingen, Germany) molecular weight separation tubes (cut-off, 20 kDa; centrifugation for 30 min at 2000 × *g*). The fraction >20 kDa was made up to a volume of 1 ml with sterile, pyrogen-free water and proteins precipitated by addition of 2 ml water-saturated phenol (10 min, 68°C). After cooling to 4°C the aqueous phase was collected and the phenolic phase again extracted with 2 ml of water. The pooled aqueous phases were centrifuged at 1 500 × *g* for 30 min and directly assayed in the LAL test in appropriate dilutions (1:200 to 1:400; test range, 0.1–1.2 EU/ml; 1 endotoxin unit equivalent to 12 ng endotoxin) without further purification. Determinations were done in duplicate in microtitre plates and false positive samples excluded from analysis. Cleavage of the S 2423 substrate was assayed at O.D. 415 nm and the endotoxin concentrations of the samples were calculated using the standard samples of the Coatest kit. The cut-off value (mean value ± 2 S.D.) for positive detection of ET was 10 EU/ml in a group of 18 liver graft recipients without complications at control examinations.

*Measurement of tumor necrosis factor-α:* TNF-α was determined with an immunoradiometric assay kit consisting of precoated capture and tracer monoclonal antibodies (TNF-α–IRMA, Medgenix Diagnostics, Brussels, Belgium). The test was carried out according to the manufacturer's instructions using plasma samples in duplicate (200 μl each). The detection limit of this method is 5 pg TNF-α/ml and the test range was 5–5000 pg/ml. Normal values were 5 ± 3.5 pg TNF-α/ml in healthy controls (mean ± S.D., $n = 16$).

*Measurement of interleukin-6:* IL-6 was determined with an ELISA-kit (Quantikine IL-6; Research and Diagnostics System, Minneapolis, MN) with a precoated monoclonal catcher antibody and an enzyme-linked polyclonal antibody preparation for detection of bound IL-6. The detection limit is 5 pg IL-6/ml and the test range 5–2000 pg/ml. No IL-6 could be detected in plasma samples from healthy control persons.

*Statistical analysis:* Differences in mean cytokine plasma concentrations between different LTX groups were analysed by Student's *t*-tests.

### Results

*Intraoperative plasma concentrations of endotoxin:* Samples containing more than 10 EU/ml endotoxin were regarded as ET positive. According to their intraoperative courses of ET the graft recipients were assigned to one of four groups. The patients found to be ET positive at the beginning of the transplantation ($n = 12/38$) were further subdivided in two groups, one remaining ET positive for all further determinations (group 1, $n = 6/12$) and the other turning ET negative at the end of grafting (group 2, $n = 6/12$). Of the pre-operatively ET negative patients, 5/26 recipients became ET positive intraoperatively (group 3) and 21/26 were again ET negative at the end of the operation, showing a transient increase (15.9 ± 7.2 EU/ml) in circulating ET during the anhepatic phase in half of the cases (Fig. 1). In ET positive patients (group 1), the ET concentrations were further increased during the anhepatic phase (C–D), dropped upon recirculation (E) and returned to pre-operative positive levels at the end of the operation (F; Fig. 1). In patients turning ET positive, the continuous increase in ET started in the early phase (B) of liver transplantation and for the two remaining groups (3 and 4) the highest ET concentrations were found at the end of the anhepatic phase (observation D).

*Intraoperative plasma concentrations of TNF-α and IL-6:* For TNF-α 6/38 patients were positive (>100 pg/ml) pre-operatively and exhibited a doubling of their TNF-α plasma concentrations during liver transplantation (Fig. 2A, group 1), 10/38 recipients became TNF-α positive until the end of grafting (group 2), and the remaining patients (22/38) were TNF-α negative throughout the operation. As

FIG. 1. Intraoperative course of endotoxin (ET) in four subgroups of liver transplant recipients ($n = 38$), classified according to their ET plasma concentrations at the beginning (A) and end (F) of grafting. Group 1 ($n = 6$), +A +F; group 2 ($n = 6$), +A −F; group 3 ($n = 5$); −A + F; and group 4 ($n = 21$), −A −F. Blood samples were collected intraoperatively after incision of the skin (B), at the beginning of the anhepatic phase (C), at the end of the anhepatic phase (D), 5 min following recirculation (E) and at the end of operation (F). Mean values (±S.E.M.) are given as endotoxin units (EU). ■, Group 1; ▨, group 2; □, group 3; ▩, group 4.

FIG. 2. (A): Intraoperative course of TNF-α in patients with permanent elevations (TNF-1; $n = 6$), patients with intraoperative induction of TNF-α (TNF-2; $n = 11$) and TNF-α negative patients (TNF-3; $n = 21$) during liver transplantation. Mean values (±S.E.M.) are shown. Samples were collected at the beginning of the operation (A), after incision of the skin (B), at the beginning of the anhepatic phase (C), at the end of the anhepatic phase (D), 5 min following recirculation (E) and at the end of grafting (F). The differences between groups are significant ($p < 0.05$) for all observations for TNF-1, and for E–F for TNF-2 and TNF-3. (B): Intraoperative course of IL-6 in patients with permanent elevations (IL-6/1, >800 pg/ml; $n = 4$), intraoperative induction of IL-6 (IL-6/2, $n = 12$) and IL-6 negative recipients. Mean values (±S.E.M.) are shown. The differences between the IL-6/2 and IL-6/3 groups are significant ($p < 0.02$) for D–F.

published previously, the TNF-α elevation became apparent at the initiation of the recirculation (Fig. 2A, group 2, E).[7] IL-6 was found to be elevated (>800 pg/ml) pre-operatively in 4/38 patients (Fig. 2B, group 1), to show increases in 12/38 patients (group 2) and to remain negative in 22/38 patients (group 3) during liver grafting.

*Intraoperative TNF-α and IL-6 induction in the different endotoxin subgroups:* The relationship of ET to TNF-α and IL-6 variations during liver transplantation are shown in Table 1 for the individual patients. In the endotoxin positive subgroup (group 1) TNF-α and IL-6 are detectable at high plasma levels in most patients. This group consists of patients with fulminant hepatitis, post-hepatic cirrhosis, a case of retransplantation with infectious complications and three cases of end-stage cirrhosis. For patients turning endotoxin negative during transplantation (group 2), 4/6 patients showed elevated TNF-α plasma levels, with parallel changes in IL-6 in the minority of these patients. In the case of induction of endotoxin during LTX (group 3), TNF-α is detectable in some, but not all patients, which in addition show IL-6 elevations in most cases, and in the endotoxin negative recipients 6/21 exhibited elevated TNF-α concentrations, with parallel increases in IL-6 in half of the cases.

**Table 1.** Intraoperative courses of TNF-α and IL-6 in individual liver graft recipients, grouped according to their endotoxin plasma concentrations at the beginning and end of operation

| Endotoxin | TNF-α | IL-6 | Concordant |
|---|---|---|---|
| Group 1 (+ → +)<br>($n = 6$) | + + +<br>elev<br>– – | + + +<br>elev<br>elev – | 83% |
| Group 2 (+ → –)<br>($n = 6$) | +<br>3 × elev<br>– – | +<br>elev – –<br>– + | 33% |
| group 3 (– → +)<br>($n = 5$) | + +<br>elev<br>– – | + +<br>elev<br>– elev | 60% |
| group 4 (– → –)<br>($n = 21$) | + + + + +<br>elev<br>15 × – | + – – – –<br>–<br>+ + + +<br>elev<br>10 × – | 52% |

The courses of TNF-α and IL-6 are symbolized for each patient in corresponding order; recipients with TNF-α > 100 pg/ml and with IL-6 > 800 pg/ml at the end of operation (time point F) were classified as positive; elevated denotes the patients with permanent intraoperative elevations and the degree of parallel changes in the cytokines under investigation for individual patients is shown as percentage of concordance.

*Correlation of early post-operative complications with intraoperative ET, TNF-α and IL-6:* Endotoxin was detectable in 5/11 patients with rejections, 4/7 patients with infections, 2/2 patients with both complications and in 6/21 patients with uncomplicated post-operative course. For the latter group, in 4/6 patients the ET disappeared from the systemic circulation during liver grafting. With exception of two patients all recipients with elevated intraoperative TNF-α concentrations subsequently rejected their grafts. Furthermore TNF-α was found in one case of infection and in one case without complications. In two cases, rejection of the grafts was found without preceding intraoperative TNF-α elevations. IL-6 was elevated in 15/38 patients, 6/8 infections, 3/13 rejections and in 6/21 patients with uncomplicated course.

### Discussion

The sequence of events leading ultimately to allograft destruction has not been fully characterized. The authors have shown that in human orthotopic liver transplantation graft rejection in the first 2 weeks is preceded intraoperatively by the appearance of TNF-α in the majority of the respective recipients.[7,8] In addition, intraoperative elevations of IL-6 plasma concentrations were correlated with early post-operative infectious complications.[9] These findings are supported by reports describing the appearance of TNF-α during rejections and the efficacy of anti-TNF antibodies in its prevention and reversal.[5,6]

In this study the possible mechanisms of TNF-α induction during human liver grafting were investigated by relating the intraoperative course of this cytokine to that of circulating ET and IL-6 in individual patients. Clinical and experimental liver transplantation are known to induce intraoperative systemic endotoxaemia and, since ET is a highly potent inducer of TNF-α and other mediators in Kupffer cells, alveolar macrophages and monocytes, this mechanism was held responsible for the induction of these cytokines, apparently operative in the pathogenesis of liver/lung injuries and early graft failures.[4,5] This model of ET induced TNF-α production is contradicted for clinical liver transplantation by our previous findings, failing to detect a direct correlation between intraoperative elevations in ET and specific post-operative complications.[7]

In an extension of the earlier studies, we have therefore tried to analyse the detailed time courses and correlations of ET and cytokine patterns in individual patients in a group of 38 liver graft recipients. According to their ET plasma concentrations at the beginning and the end of transplantation the recipients were assigned to the ET negative, ET positive for all intraoperative observations, ET disappearing and ET induced subgroups. The cut-off value for ET was 10 EU/ml, as defined in LTX outpatients without complications, and the 'ET negative' subgroup included patients with low and transient ET elevations during the anhepatic phase. The primarily ET positive recipients included patients with acute liver failure (fulminant hepatitis, graft failure) and advanced stages of cirrhosis, showing TNF-α and IL-6 elevations in most cases. Interestingly, six ET positive recipients (one case of Wilson's disease and five cases with cirrhosis) turned ET negative during grafting and four of them showed no further signs of complication in the post-operative phase, indicating permanent removal of ET by the grafted liver. Five patients, again with acute liver failure and advanced cirrhosis, turned ET positive and 21/38 patients exhibited no or only transient and moderate intraoperative ET elevations.

The recipients with pre-transplant elevations of ET showed permanent or induced TNF-α elevations in the majority of the cases (8/12) and in patients turning ET positive intraoperatively, 3/5 showed increases in TNF-α. Five out of 21 ET negative patients showed elevations in TNF-α (observation E, > 100 ng/ml) in the absence of any transient increases of ET during the anhepatic phase, indicating non-ET associated induction of TNF-α. As described previously the TNF-α positive recipients showed a high rate of early post-operative rejections (85%; 11/13 patients), suggesting that TNF-α may be an important predisposing factor in alloantigen reactivity, independent of its mode of induction. Although TNF-α may induce rejection by several direct acting mechanisms, including promotion of leucocyte adherence or stimulation of MHC antigen expression in the allograft,[11,12] our data cannot exclude indirect effects of TNF-α on the induction of further cytokines. Intraoperative elevations in IL-6 were detected in 15/38 patients with 6/15 post-operative infectious complications. IL-6 seems to precede the elevations in TNF-α and its course parallels that of TNF-α in about half of the patients (21/38), indicating a TNF-α-independent regulation in the remaining recipients.

In summary, the liver graft recipients exhibit complex patterns of cytokine expression. A group of patients showed pretransplant elevations of ET due to the complications associated with the underlying end-stage liver diseases, accompanied by permanent production of TNF-α and a high incidence of early graft rejections. Non-cleared ET at the end of grafting was associated with TNF-α elevations, whereas the transient ET increases during the anhepatic phase did not result in subsequent significant TNF-α elevations and clinical complications. Apparently non-ET mediated TNF-α induction was detected in a sub-group of patients showing a high frequency of graft rejection. Maximal TNF production of macrophages/monocytes in response to endotoxin/alloantigens needs approximately 6 h and is apparently too slow to account for the observed increases during the final stages of human liver transplantation. Since TNF-α was elevated after liver transplantation between syngeneic inbred rats, the liver conservation/transplantation procedure itself seems to be a cause of elevated TNF-α.[5] In clinical transplantation increased rates of rejections were reported in patients with preservation injuries of their grafts[13] and fulminant hepatitis.[14] The ET independent TNF-α production during organ preservation/reperfusion is likely to represent a mediator of graft inflammation after recirculation. We failed to detect IFN-γ or IL-4 in plasma samples from eight liver recipients (IFN-γ RIA and IL-4 ELISA), but local production and interaction of several cytokines in TNF-α positive patients is likely to occur during initiation of graft rejection. Prophylactic inhibition of TNF-α by specific antibodies or inhibitors (like soluble TNF receptors) initiated prior to allograft exposure may help to prevent the early events leading to immunological responses and graft destruction as well as lung/liver .injury caused by this cytokine during grafting.

### References

1. Starzl TE, Demetris AJ, Van Thiel DH. Liver transplantation. *N Engl J Med* 1989; 321: 1092–1099.
2. Quiroga J, Colina I, Demetris AJ, Starzl TE, Van Thiel DH. Cause and timing of first allograft failure in orthotopic liver transplantation: a study of 177 consecutive patients. *Hepatology* 1991; **14**: 1054–1062.
3. Kraus TH, Noronha IL, Manner M, Klar E, Küppers P, Otto G. Clinical value of cytokine determination for screening, differentiation, and therapy monitoring of infections and noninfectious complications after orthotopic liver transplantation. *Transplant Proc* 1991; **23**: 1509–1512.
4. Yokoyama I, Todi S, Miyata T, Selby R, Tzakis AG, Starzl TE. Endotoxemia and human liver transplantation. *Transplant Proc* 1989; **21**: 3833–3841.
5. Goto M, Takei Y, Kawano S, *et al.* Tumor necrosis factor and endotoxin in the pathogenesis of liver and pulmonary injuries after orthotopic liver transplantation in the rat. *Hepatology* 1992; **16**: 487–493.
6. Imagawa DK, Millis JM, Olthoff KM, *et al.* The role of tumor necrosis factor in allograft rejection. *Transplantation* 1990; **50**: 219–225.
7. Hamilton G, Prettenhofer M, Zommer A, *et al.* Intraoperative course and prognostic significance of endotoxin, tumour necrosis factor-α and interleukin-6 in liver transplant recipients. *Immunobiol* 1991; **182**: 425–439.
8. Függer R, Hamilton G, Steininger R, Mirza D, Schulz F, Mühlbacher F. Intraoperative estimation of endotoxin, TNF-α, and IL-6 in orthotopic liver transplantation and their relation to rejection and post-operative infection. *Transplantation* 1991; **52**: 302–306.
9. Hamilton G, Tüchy G, Hamilton B. Intraoperative kinetics and regional distribution of interleukin-6 (IL-6) during human liver transplantation. *Transplantation* 1992; **54**: 746–748.
10. Berger D, Marzinzig E, Marzinzig M, Beger, HG. Quantitative endotoxin determination in blood—chromogenic modification of the Limulus amoebocyte lysate test. *Eur Surg Res* 1988; **20**: 128–136.
11. Munro JM, Pober JS, Contran RS. Tumour necrosis factor and interferon induce distinct patterns of endothelial activation and associated leukocyte accumulation in skin of papioanubis. *Am J Pathol* 1989; **135**: 121–133.
12. Halloren PF, Wadgymar A, Autenried P. The regulation of expression of major histocompatibility complex products. *Transplantation* 1986; **41**: 413–420.
13. Howard TK, Klintmalm GB, Cofer JB. The influence of preservation injury on rejection in the hepatic transplant recipient. *Transplantation* 1990; **49**: 103–107.
14. Mor E, Solomon H, Gibbs JF, *et al.* Acute cellular rejection following liver transplantation: clinical pathological features and effect on outcome. *Semin Liver Dis* 1992; **12**: 28–40.

ACKNOWLEDGEMENTS. We thank Ms M. Prettenhofer and Ms H. Zommer for excellent technical assistance and all members of the transplant unit and the intensive care unit for supporting this investigation.

**Received 20 April 1993; accepted in revised form 17 May 1993**
